# Analysis of *t-*test misuses and SPSS operations in medical research papers

**DOI:** 10.1186/s41038-019-0170-3

**Published:** 2019-11-08

**Authors:** Guangping Liang, Wenliang Fu, Kaifa Wang

**Affiliations:** 1 State Key Laboratory of Trauma, Burns, and Combined Injury, Institute of Burn Research, Southwest Hospital, Third Military Medical University (Army Medical University), Chongqing, 400038 People’s Republic of China; 2 School of Mathematics and Statistics, Southwest University, Chongqing, 400715 People’s Republic of China

In medical research papers, the selection of appropriate statistical methods serves as one of the pivotal premises to ensure the quality of papers and credibility of their results [1–3]. To correctly perform the statistical analysis of quantitative data, two key points should be considered. One is to identify the type of experimental design correctly, and the other is to check whether data meets the preconditions of parameter test [2–4]. Otherwise, it may cause different misuse in some situations and may even draw different or opposite conclusions about the same data.

As one of the most commonly used statistical methods in medical research papers, *t-*test can be divided into one-sample *t-*test and two-sample *t-*test [3, 4]. Thus, it is inappropriate to compare the means among multiple groups (more than three). Concretely, one-sample *t-*test is used to compare one group’s average value to a single number (a known population mean, for example, the norm). The two-sample *t*test is a type of inferential statistic used to determine if there is a significant difference between the means of two groups. Furthermore, there are two types of two-sample *t*-test [3, 4]. One is independent sample *t-*test (group *t-*test), which is performed when the samples typically consist of independent population. The other is paired (or correlated) sample *t-*test, which is used when each observation in one group is paired with a related observation in the other group, i.e., the samples typically consist of matched pairs of similar units, or when there are cases of repeated measures.

Note that *t-*test belongs to the category of parametric test. The assumptions of the parametric test, including independence, normality, and homogeneity of variance, must be met to ensure the correct use of *t-*test [3, 4]. In addition, according to the theoretical deduction of *t-*test, it can only be applied to the quantitative data of single factor design, so it is inappropriate to perform *t-*test for multifactor design. For example, there are multiple independent variables/factors (such as gender and different types and dosage of drugs) and the comparisons among groups after controlling for simple effects of each independent variable.

As a journal editor and reviewer, we often encounter that some authors blindly use *t-*test to process quantitative data without analyzing the prerequisites of *t-*test or considering the type of experimental design, especially to independent sample *t-*test (group *t-*test). In order to improve the quality of statistical analysis in medical research papers, according to the problems found in the process of reviewing manuscripts, we summarized the following five most common misuses of *t-*test and analyzed them with examples. We hope that it can provide real help to improve our data analysis ability.

It is particularly noted that all the examples herein are artificially constructed for the purpose of illustration and do not represent actual clinical design and data. They are only for reference in the selection of statistical analysis methods.

## Misuse of *t-*test because data do not obey normal distribution

Normal fitting tests, including the Shapiro-Wilk test for small sample size (*n* ≤ 50) or Kolmogorov-Smirnov test for large sample size (*n* > 50), usually require the analysis of the original data. However, there is a common and concise method to judge whether the data obey normal distribution, that is, to compare the mean and corresponding standard deviation (SD) of the data. If the mean is much smaller than its standard deviation, then the data may not obey the normal distribution, so *t-*test may also be inappropriate. In this case, it is better to perform *t-*test after an appropriate variable transformation (such as logarithm transforms and rank transforms) or perform nonparametric test method for original data.

### Example 1

A researcher adopts the independent sample *t-*test to compare the demographic data (age) between the experimental group and the control group. Table 1 provides the statistical results (see Additional file 1 for the original data). Is this appropriate?

**Table 1:** Statistical results of age between experimental group and control group

Group n	Age (years)	*t* value	*P* value
Experimental group 20	21.30b ± 8.39	0.050	0.961
Control group 20	21.60 ± 25.49		

Note: Data are presented as mean ± standard deviation

### [Analysis]

The data are quantitative data for two independent samples under single factor design. However, from Table 1, we can find that the standard deviation is larger than its mean value in control group. Thus, the age in control group may not meet normal distribution. As a result, it may be inappropriate to analyze this data by the independent sample *t-*test directly.

### [Correction]

Since the sample size of two groups is less than 50, the Shapiro-Wilk test is more suitable for normal fitting test. Selecting “Analyze➔Descriptive Statistics➔Explore…” and ticking “Normality plots with tests” in the “Plots” dialog box in SPSS. The results show that the age in experimental group accepts the normal distribution hypothesis (*W* = 0.915, *p* = 0.080), but the age in control group rejects the normal distribution hypothesis (*W* = 0.635, *p* < 0.001). Therefore, appropriate variable transformation should be performed if *t-*test must be used. In fact, the nonparametric Wilcoxon rank sum *W* test is a simpler and more suitable statistical method, and the Mann-Whitney *U* test method should be selected in this case. Selecting “Analyze➔Nonparametric Tests➔2 Independent Samples…” and ticking “Mann-Whitney *U*” in “Test Type” part. After performing the test in SPSS, we have the test statistic *U* = 116.500 and *p* = 0.024. As a result, we can conclude that the difference of mean rank has statistical significance between the experimental group and control group, which is completely contrary to the results of independent sample *t-*test (Table 1). By the way, when a variable does not obey the normal distribution, it is better to report as median with its corresponding first and third quartiles (Q1–Q3) or median with its range, not as mean and standard deviation. In the following parts, all variables are subject to the assumption of normal distribution without special explanation.

## Misuse of independent sample *t-*test because of paired samples

In medical research, before-after study in the same patient is often used to compare the effect of a treatment factor (such as drug and operation). This is a typical self-matching experimental design type, which does not meet the independent assumption of independent sample *t-*test. In this case, the paired sample *t-*test is more suitable if the difference value is met normally distributed. Otherwise, the nonparametric test (Wilcoxon signed rank test) of two related samples is recommended.

### Example 2

In order to explore the effect of a certain treatment scheme on the scar of burn patients, the scar area of the patients is measured 1 day before and 1 week after treatment, respectively. And the independent sample *t-*test is used to compare the changes of scar area of the patients before and after treatment. Table 2 shows the statistical results (see Additional file 2 for the original data). Is this appropriate?

**Table 2:** Evaluation of scar area of burn patients before and after treatment

Group n	Scar area (cm^2^)	*t* value	*P* value
Before treatment 20	5.38 ± 1.64	2.695	0.010
After treatment 20	4.05 ± 1.47		

Note: Data are presented as mean ± standard deviation

### [Analysis]

Clearly, the independent assumption of independent sample *t-*test is not satisfied under the study protocol, and independent sample *t-*test is inappropriate for the data.

### [Correction]

Using the origin data and paired samples *t-*test, i.e., selecting “Analyze➔Compare Means➔Paired-Samples T Test…” in SPSS, we have the test statistic *t* = 10.025 and *p* < 0.001. It should be noted that in this example, by comparing the *p* values obtained by the two methods, we find that the result of the independent sample *t-*test may underestimate the efficacy of the treatment scheme, though both results indicate that the treatment scheme can significantly reduce the scar area of burn patients.

## Misuse of independent sample *t-*test because there are more than three levels in independent samples

The single factor *k*-level (*k* ≥ 3) independent sample design is a widely used experimental design method in medical experiments. For example, to investigate the difference of a physiological index with different disease types, we measured the index of patients with *k* (*k* ≥ 3) disease types. In this case, we need to compare the means among *k* independent samples and determine whether there are any statistically significant differences between the means of three or more independent (unrelated) groups. Because direct multiple use of independent samples *t-*test will increase the probability of type I error, one-way analysis of variance (ANOVA) is more suitable at this time. If the one-way ANOVA returns a statistically significant result, we accept the alternative hypothesis, which is that there are at least two group means that are statistically significantly different from each other. To determine which specific groups differed from each other, we further need to perform post hoc multiple comparisons. If we want to compare each group with the control group, Dunnett’s test is recommended.

### Example 3

For a new antihypertensive drug, we hope to compare the antihypertensive effect of high- and low-dose groups with that of placebo group. The independent sample *t-*test is adopted to compare the low-dose group with the placebo group and the high-dose group with the placebo group, respectively. The statistic results are presented in Table 3 (see Additional file 3 for the original data). Is this appropriate?

**Table 3:** Statistical comparison of antihypertensive effects

Group n	Decreased systolic blood pressure (mmHg)
Low-dose group 15	26.60 ± 1.765*
High-dose group 20	29.90 ± 2.404^###^
Placebo group 12	24.92 ± 1.676

Note: Data are presented as mean ± standard deviation

* means that the comparison between low-dose group and placebo group under independent samples t-test (*t* = 2.517, *p* = 0.019)

^###^ means that the comparison between high-dose group and placebo group under independent samples t-test (*t* = 6.301, *p* < 0.001)

### [Analysis]

These data are typical quantitative data of multigroup independent sample design, also known as the single factor design with multiple levels, and the number of levels is 3. Thus, it is not appropriate to perform the independent sample *t-*test directly for comparisons with control group.

### [Correction]

According to the study design, selecting “Analyze➔Compare Means➔One-Way ANOVA…” and ticking “Dunnett” in the “Post Hoc Multiple Comparisons” dialog box in SPSS, we perform one-way ANOVA and Dunnett’s post hoc test to compare each dose group with the placebo group. The results indicate that there is a statistically significant difference between groups as determined by one-way ANOVA (*F* = 24.728, *p* < 0.001). The results of multiple comparisons show that the difference between low-dose group and placebo group is not statistically significant (*p* = 0.069), which is completely contrary to the results of the independent sample *t*-test (Table 3). The difference between high-dose group and placebo group is still statistically significant (*p* < 0.001).

## Misuse of independent sample *t-*test because of factorial design data

To understand the effect of two or more independent variables upon a single dependent variable, completely randomized factorial design is often used in medical experiments or clinical trials. A factor is a variable that is controlled and varied during the course of an experiment. In a factorial design, there are two or more factors with multiple levels that are crossed, e.g., two dose levels of drug A and two levels of drug B can be crossed to yield a total of four treatment combinations. Factorial designs offer certain advantages over conventional designs. The design can examine not only the differences among the levels of each factor, but also the interactions among the factors. For quantitative data of factorial design, direct multiple use of independent sample *t-*test will not only increase the probability of type I error, but also lead to wrong conclusions when there is interaction between various factors. A more appropriate method at this point is to perform ANOVA of factorial design. Taking two factors of independent samples as an example, it is also called the two-way ANOVA of independent samples.

### Example 4

To study the difference of pain score between patients with different disease types (burn, trauma, and arthritis) after receiving two treatment schemes (named as scheme A and scheme B), ten patients were recruited for each type of disease and randomly assigned to the possible treatment schemes with equal possibility. For the measured pain scores, independent sample *t-*tests are performed repeatedly to compare the difference between disease types and treatment schemes. Table 4 shows the statistical results (see Additional file 4 for the original data). Is this appropriate?

**Table 4:** Comparison of pain scores of patients with three disease types and two treatment schemes

Disease types	*n*	Treatment schemes			
		*n*	Scheme A	*n*	Scheme B
Burn	10	5	12.00 ± 2.236	5	17.20 ± 3.194*
Trauma	10	5	20.80 ± 3.033^##^	5	10.20 ± 1.924***^,##^
Arthritis	10	5	12.80 ± 2.387^$$^	5	13.20 ± 1.924^#,$^

Note: Data are presented as mean ± standard deviation

* indicates the comparison between two treatment schemes by independent samples *t*-test, *p* < 0.05 is labeled as * and* p* < 0.001 is ***

^#^ indicates the comparison between trauma/arthritis group and burn group by independent samples *t*-test, *p* < 0.05 is labeled as ^#^ and *p* < 0.01 is ^##^

^$^ indicates the comparison between arthritis group and trauma group by independent samples t-test, *p* < 0.05 is labeled as ^$^ and *p* < 0.01 is ^$$^

### [Analysis]

This study involves two factors. One is treatment factor with two levels, scheme A and scheme B, while the other is disease type factor with three levels, burns, trauma, and arthritis. Since the patients in each level combination are different, the samples are independent. Therefore, this study belongs to the 2 × 3 factorial design, and the ANOVA of factorial design should be performed for comparative analysis. Firstly, the interaction effect between the factors should be tested. If the interaction effect is not statistically significant, the main effect of each factor can be analyzed. Otherwise, the individual effect of each factor needs to be analyzed separately.

### [Correction]

ANOVA of factorial design should be performed using the General Linear Model in SPSS (selecting “Analyze➔General Linear Model➔Univariate…”), and the results show that the interaction term between treatment and disease type reaches the significance level (*p* < 0.001), indicating that the interaction of these two factors does have an effect on the dependent variable (pain score). Therefore, it is necessary to conduct simple primary effect test for each factor. Since these two factors are independent samples, the “Split File” instruction under drop-down menu of “Data” in SPSS can be used to select qualified samples for independent sample one-way ANOVA. Through the test, in the case of scheme B, we find that there is no statistically significant difference in the pain scores between the burn/trauma and arthritis (*p* = 0.067/0.187), which is completely contrary to the results of independent sample *t-*test (Table 4).

## Misuse of independent sample *t-*test because of repeated measurement design

Repeated measurement designs are commonly used in longitudinal studies, such as the dynamic changes over time of temperature, blood pressure, and other indicators, which is often encountered in medical research. The purpose is usually to detect whether there is a statistical significance in the difference of the indicator values at different time points. In practice, many authors usually calculate the mean and standard deviation of each time point, and then carry out independent sample *t-*test repeatedly for each time point. However, according to the design principle, we know that repeated measures design uses the same subjects with every condition of the research, including the control. Thus, the measurements at different time points are correlated with each other, that is, the samples at different time points are not independent of each other. Roughly speaking, such data are often time-dependent. In this case, the appropriate analysis method is ANOVA of repeated measures designs. If there is another factor with independent samples, two-way ANOVA with mixed samples is recommended.

### Example 5

To study the difference for a certain indicator at different postoperative time points, 10 patients (5 males and 5 females) are enrolled in the study and the indictor of each of them is measured at 1, 2, 4, and 8 weeks after the operation. The researchers use the independent sample *t-*test to analyze the difference of this indictor of different time points. The statistical results are presented in Table 5 (see Additional file 5 for the original data). Is this appropriate?

**Table 5:** Comparison of a certain indicator at different postoperative time points

Gender n	Postoperative time (week)			
	1	2	4	8
Male 5	44.20 ± 4.207	35.00 ± 3.082**	19.40 ± 1.140***^,###^	37.80 ± 3.962*^,$$$^
Female 5	33.00 ± 4.183	17.00 ± 1.581***	28.40 ± 1.949^###^	37.60 ± 3.209^###,$$^

Note: Data are presented as mean ± standard deviation

* indicates the comparison between 1 week after operation and other time points by independent samples *t*-test, *p* < 0.05 is labeled as *, *p* < 0.01 is ** and *p* < 0.001 is ***

^#^ indicates the comparison between 2 week after operation and other time points by independent samples *t*-test, *p* < 0.05 is labeled as ^#^, *p* < 0.01 is ^##^ and *p* < 0.001 is ^###^

^$^ indicates the comparison between 4 week after operation and other time points by independent samples *t*-test, *p* < 0.05 is labeled as ^$^, *p* < 0.01 is $$ and *p* < 0.001 is ^$$$^

### [Analysis]

According to the experimental process of this study, the indicators of each patient are repeatedly measured at 1 week, 2 weeks, 4 weeks, and 8 weeks after the surgery, so the postoperative time serves as a factor of repeated measurement with four levels. In addition, gender is another factor, which is an independent sample at each level. Thus, the overall design was separated by pairwise comparison at different time points through independent sample *t-*test and fails to take into account the fact that the data on the same subject at different time points are not independent.

### [Correction]

Two-way ANOVA with mixed samples should be performed using the General Linear Model in SPSS (selecting “Analyze➔General Linear Model➔Repeated Measures…”). Similarly, since the interaction between gender and postoperative time reaches the level of significance (*p* < 0.001), it is necessary to perform simple primary effect test. However, since the gender factor is an independent sample and the postoperative time factor is a related sample, the test methods for the two factors are different. For gender factor, four independent sample one-way ANOVA analyses were performed based on the four levels of postoperative time, but for postoperative time factor, two related sample ANOVAs were carried out based on the two levels of gender. Using the original data, we can find that the difference between 1 week after operation and 8 weeks after operation is not statistically significant in males (*p* = 0.057), but there is a significant difference between 8 weeks after operation and 1 week after operation in females (*p* = 0.045), which was completely contrary to the results of independent sample *t-*test (Table 5).

In summary, in order to effectively reduce misuse of statistical methods and improve credibility of the statistical results, it is necessary to carefully consider the experimental design type, distribution characteristics of the data, and other relevant factors. Concretely, we should meticulously review the applicable preconditions of each statistical analysis technique and reasonably select the appropriate method before analysis of quantitative data. In this paper, the five cases of most commonly misused *t-*tests are summarized, with the causes of each misuse analyzed and the more appropriate statistical methods are also offered in SPSS. By doing so, we believe that this paper can be helpful to the writing and editing of biomedical research papers.

## Supplementary information

**Additional file 1:** The original data of the Example 1.

**Additional file 2:** The original data of the Example 2.

**Additional file 3:** The original data of the Example 3.

**Additional file 4:** The original data of the Example 4.

**Additional file 5:** The original data of the Example 5.

## Data Availability

All artificially constructed data are presented in the tables and additional files.

## Abbreviations

ANOVA: Analysis of variance
SD: Standard deviation
